# The Meeting of the Association

**Published:** 1893-05

**Authors:** 


### *EDITORIAL.*

---

The office of The Journal is in room 330 Equitable Building.

Address communications relating to the Editorial Department to Dr. L. B. Grandy, Atlanta, Ga., Box 431.

Business communications should be addressed to Dr. M. B. Hutchins, Atlanta, Ga. Box 431.

All remittances should be made payable to "Atlanta Medical and Surgical Journal."

Articles for publication should reach this office not later than the 15th instant.

The editors are not responsible for views expressed by contributors.

---

### THE MEETING OF THE ASSOCIATION.

---

The meeting at Americus was probably the most successful one in recent years. While we noted the absence of many prominent men whose presence and co-operation would have been desirable, the meeting was well attended and many papers of a high order of merit presented and discussed. We were pleased to notice that the members in attendance with great unanimity seemed to realize that something had to be done to improve the condition of the association. To eliminate the political spirit which has exerted a bad influence in late years a change was made in the manner of electing officers.

Just as the counties were selecting their delegates to represent them in the Nominating Committee, Dr. W. F. Westmoreland introduced a resolution to strike out sections 11 and 15 of the by-laws, referring to Nominating Committee, and to substitute "all elections to be held by ballot, without nomination, on the morning of the last day of the meeting." This was carried by the requisite two-thirds vote.

As the Nominating Committee had been formed the President *pro tem.* was inclined to rule that the new by-law did not take effect until the next meeting, 1894, and wished to read the list of the Nominating Committee who would meet Thursday evening, as heretofore, and make nominations. A motion by Dr. J. G. Hopkins, of Thomasville, to adjourn, was carried and the Nominating Committee became *non est.*

The necessity of a State Board of Examiners was brought before the association, both in the President's address and in a paper which appears in this issue of THE JOURNAL. The matter of a State Board of Health was also presented and discussed. The better part of the profession, we believe, will be pleased to learn that the association planted itself squarely on these issues, and appointed a committee (one member from each congressional district) to convey its memorial to the next meeting of the legislature. We have no doubt that this will secure the needed legislation, and that a Board of Examiners will be established in the fall, the efforts of quacks, eclectics, lawyers and others to the contrary, notwithstanding.

Concerning certain forms of advertising, which, it appears, have been practiced by some members, the association took important action. This related to the placing of professional cards about hotels, and inserting or causing to be inserted in the papers, notices of operations, cures, etc. Resolutions were adopted declaring such practice to be highly reprehensible, contrary to our ethics, and promising to deal with future offenders as circumstances indicate.

During the session of Friday morning the election of officers was taken up. The ballots were prepared, without nominations, and tellers received them.

Dr. W. H. Elliott, of Savannah, received 60 votes.

Dr. M. H. O'Daniel, of Macon, received 22 votes.

Scattering, 8 votes.

Dr. O'Daniel made an eulogistic speech in favor of making the election of Dr. Elliott unanimous. Upon his motion, Dr. Elliott was unanimously elected by a *viva voce* vote. Dr. Westmoreland immediately moved that the election of Dr. Elliott be further made unanimous by a rising vote. Carried. Upon the vote being called for by the President, the body rose *en masse*. Dr. Elliott thanked the association for the honor. He expected good to come to the association from the resolutions made by the members.

The new by-law had a chance to be enforced just here. The election of Vice-Presidents being in order, Dr. M. H. O'Daniel began a speech in favor of Dr. Miller, of Americus, for First Vice-President. Upon a point of order by Dr. Fitts, of Carrollton, the President ruled that no nomination could be made. The counting of the ballots showed the election of Dr. G. T. Miller (Chairman Committee of Arrangements), of Americus, as First Vice-President. Dr. H. McHatton, of Macon, was elected Second Vice-President.

Under a precedent established when Dr. K. P. Moore was elected Secretary, it was decided that Dr. Dan Howell should continue as Secretary for his full five years.

An eloquent oration upon "Woman's Relations to the Practice of Medicine" was delivered by Dr. Frank Ridley at 12 M.

The following of our advertisers had exhibits at Americus:

Tarrant & Co., with the only genuine *Hoff's Malt Extract* and *Seltzer Aperient*. Mr. N. E. Hulbert in charge.

Doliber-Goodale Co., with their unrivaled *Mellin's Food.* Mr. C. H. Robins was in charge and had that picture of the Mellin's Food baby (one of them) everywhere.

Messrs. Renz & Henry, with their excellent *Three Chlorides* and *Tri-Iodides*. Dr. D. W. Whittaker, their courteous representative, in charge.

The New York Pharmacal Association and the Arlington Chemical Co., makers of the finest of their kind of preparations. The handsome C. H. Pearson was in charge.

Reed & Carnrick, with their well and favorably known preparations were also represented.

Members got all the samples they wanted and should get all the *results* they want.

Paul Herth represented Wyeth & Bro., M. Bartlett had Appleton's books, E. A. Yarnall Co. had instruments, Mr. Jones had the Columbia Cyclopedia, J. L. Wohlgemuth had Malted Milk, W. H. Campbell showed Saunders' medical publications, Warner & Co. samples.

EXIT *politics;* enter good old members who wouldn't attend in company with *politics.*

NOW we need a by-law rendering any member ineligible to office who shall electioneer for it in any way.

THE size of the crowd rather paralyzed the Hotel Windsor. It is a fine hotel, *but the crowd was too much.*

IT will not be so easy to "pack" a whole association as to "pack" a Nominating Committee; and *politics* got a black eye.

AFTER awhile THE JOURNAL can't get any new subscribers in the State Medical Association. Why? Because it has nearly all of them now.

THE exhibitors presented Dr. Miller with a handsome gold-headed cane in appreciation of his courtesies as Chairman of the Committee of Arrangements.

THE newspapers were rather "fresh" in their reports of a "row" between two of the members. It didn't amount to anything, and everything was smooth in a moment.

DR. RICHARDSON, of Montezuma, made an eloquent and convincing speech in favor of abolishing the Nominating Committee, and no doubt aided much in the new by-law being adopted.

ELECTIONS should be held on Thursday evening of the meeting, between the supper hour and the hour for the banquet. Let that also be the time for distributing tickets to the banquet.

THE JOURNAL would like to make personal mention of each one of its friends at the association, but really, it would take too many pages. So we herewith express our compliments and good wishes to them all.

LET every member set his plans to be in Atlanta at the next meeting, and bring a few new members with him. We promise you a pleasant meeting and THE JOURNAL'S best efforts to make you enjoy yourself.

